# Prognostic value of NT-proBNP monitoring in patients with left ventricular assist devices

**DOI:** 10.1016/j.jhlto.2025.100387

**Published:** 2025-09-20

**Authors:** Miloud Cherbi, Joaquim Verdaguer, Romain Itier, Laurence Barde, Pauline Fournier, Etienne Grunenwald, Philippe Gaudard, Philippe Rouvière, Adrien Molina, Aurore Ughetto, Clément Delmas

**Affiliations:** a Department of Cardiology, Toulouse University Hospital, Toulouse, France; b Department of Cardiac Surgery, Toulouse University Hospital, Toulouse, France; c Department of Anesthesiology and Critical Care Medicine, Arnaud de Villeneuve Hospital, CHU Montpellier, University of Montpellier, Montpellier, France; d Department of Cardiac Surgery, CHU Montpellier, University of Montpellier, Montpellier, France; e Toulouse University, Toulouse, France; f Recherche et Enseignement en Insuffisance Cardiaque Avancée Transplantation et Assistance Cardiaque (REICATRA), Institut Saint Jacques, Toulouse, France

**Keywords:** advanced heart failure, LVAD, BNP, NT-proBNP, monitoring, outcomes

## Abstract

**Background:**

N-terminal fragment of the brain natriuretic peptide (NT-proBNP) is a well-established biomarker in heart failure; however, its prognostic value in patients supported by left ventricular assist devices (LVAD) remains unclear.

**Methods:**

We conducted a retrospective cohort study including patients implanted with an LVAD between 2008 and 2025 at 2 tertiary care centers. NT-proBNP levels were measured at 3 and 6 months postimplantation, and their relationships with invasive hemodynamic parameters and 1-year clinical outcomes were evaluated at each time point.

**Results:**

A total of 128 patients were included. Whereas NT-proBNP measured at 3 months showed no significant correlation with any hemodynamic parameters, NT-proBNP at 6 months correlated with right atrial pressure (ρ = 0.46, *p* < 0.01), systolic pulmonary artery pressure (ρ = 0.39, *p* = 0.03), and pulmonary capillary wedge pressure (ρ = 0.39, *p* = 0.02). NT-proBNP measured at 3 months was not significantly associated with 1-year mortality, pump thrombosis, or right ventricular (RV) failure. Conversely, NT-proBNP at 6 months was independently associated with an increased risk of the composite end-point of mortality or pump thrombosis (adjusted hazard ratio 1.34 [1.07-1.62] per 500 pg/ml increase), and the composite end-point of mortality or RV failure (adjusted odds ratio 1.53 [1.17-2.01] per 500 pg/ml increase).

**Conclusions:**

NT-proBNP measured at 6 months post-LVAD implantation is a significant predictor of adverse clinical and hemodynamic outcomes at 1 year, supporting its role in risk stratification. Prospective studies are warranted to validate these findings.

## Graphical Abstract

Central illustration: Prognostic value of NT-proBNP monitoring in patients with left ventricular assist devices. ACE-I, angiotensin-converting enzyme; ARB, angiotensin II receptor blockers; ARM, aldosterone receptor antagonists; ARNi, angiotensin receptor neprilysin inhibitor; BTT, bridge-to-transplant; CI, cardiac index; DT, destination therapy; LVAD, left ventricular assist device; PAP, pulmonary artery pressure; PAPI, pulmonary artery pulsatility index; PCWP, pulmonary capillary wedge pressure; RV, right ventricular.

**Figure:**
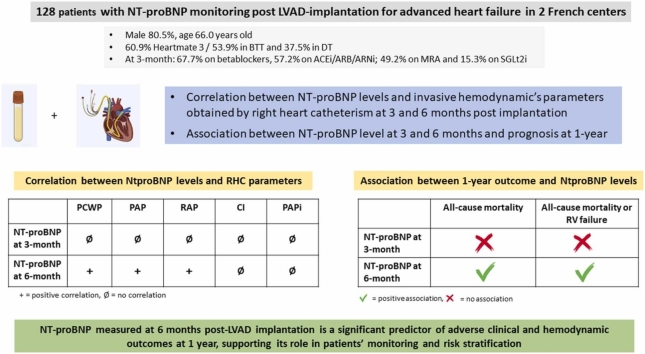

## Background

As advanced heart failure (HF) becomes increasingly prevalent, left ventricular assist devices (LVAD) have emerged as a cornerstone therapeutic option. Offering significant improvements in both survival and quality of life, LVAD are now routinely employed as bridge-to-transplant (BTT) or destination therapy (DT), with current guidelines granting a class IA recommendation for INTERMACS profiles 1 to 3 and class IIb-B for profile 4.^1^ Due to successive technological advancements, patients supported with the latest-generation device—HeartMate 3 (Abbott, Abbott Park, IL)—now achieve a median survival approaching 6 years.^2^ However, this remains markedly lower than the median survival after heart transplantation, which exceeds 10 years.^3^ In LVAD recipients, long-term outcomes are heavily influenced by device-related complications such as bleeding, right ventricular (RV) failure, and infections.^4^, ^5^ As such, optimizing the management of LVAD patients remains a major challenge, especially in the current landscape of rising advanced HF burden and persistent donor shortage.^6^, ^7^

While the prognostic value of N-terminal fragment of the brain natriuretic peptide (NT-proBNP) has been extensively validated in HF patients without LVADs—being closely linked to both survival and quality of life^8^, ^9^—its utility in the context of LVAD support remains poorly understood. In native hearts, NT-proBNP is secreted in response to increased cardiac wall stress and serves as an easily accessible biomarker to monitor HF progression in both acute and chronic settings.^10^ It is also well correlated with key hemodynamic parameters of pressure and flow from both ventricles.^11^ Whether NT-proBNP can serve a similar role in the follow-up of LVAD patients remains unknown but could represent a valuable, noninvasive tool to improve long-term care.

Therefore, this study aimed to evaluate the prognostic value of NT-proBNP in patients with LVAD, focusing on both clinical and hemodynamic outcomes.

## Methods

### Study design

The present study was conducted at 2 tertiary LVAD centers in France (University Hospital of Toulouse and University Hospital of Montpellier). All patients who underwent durable LVAD implantation at these institutions between January 1, 2008, and January 1, 2025, were consecutively included in a retrospective registry with standardized data collection procedures. Eligible patients were adults aged 18 years or older who received either an HVAD (HeartWare, Minnesota, MN), HeartMate II (Abbott, Pleasanton, CA), or HeartMate III (Abbott, Abbott Park, IL) and had a recorded NT-proBNP measurement at 3 months postimplantation. Patients were excluded if they died within the first 3 months following LVAD implantation or had a history of prior LVAD implantation or heart transplantation.

### Baseline assessment and follow-up

Preimplantation data were retrieved from hospital records for all included patients and comprised demographic characteristics, cardiovascular risk factors, comorbidities such as chronic obstructive pulmonary disease, chronic kidney disease (CKD), and prior stroke, as well as underlying cardiac and HF etiologies, prior medications, history of supraventricular or ventricular arrhythmias, laboratory findings, echocardiographic parameters—including left ventricular ejection fraction (LVEF)—and functional status assessments such as New York Heart Association classification, peak oxygen uptake, and the 6-minute walk test (6MWT). Patient monitoring was carried out according to the protocols specific to each participating center. Follow-up visits took place at 3, 6, and 12 months postimplantation and included clinical evaluations, laboratory tests, and echocardiographic assessments. Right heart catheterization was performed based on the treating physician’s judgment and the patient’s clinical evolution. All right heart catheterization procedures were conducted by experienced HF physicians. Hemodynamic measurements were recorded at end-expiration and included right atrial pressure (RAP); systolic, mean, and diastolic pulmonary artery pressures (sPAP, mPAP, and dPAP); pulmonary capillary wedge pressure (PCWP); and thermodilution cardiac output. Pulmonary artery pulsatility index (PAPI), cardiac index (CIx), and the RAP/PCWP ratio were further calculated.

### End-points

The primary end-point was all-cause mortality at 1 year. Secondary clinical outcomes included the composite of all-cause mortality or pump thrombosis, and the composite of all-cause mortality or late RV failure, both assessed at 1 year. Hemodynamic outcomes comprised RAP, sPAP, mPAP, dPAP, PCWP, cardiac output, PAPI, CIx, and the RAP/PCWP ratio, all assessed at 3 and 6 months.

### Statistical analysis

Quantitative variables are reported as medians and interquartile ranges (IQR), while categorical variables are described as frequencies and percentages. Comparisons between groups were performed using the Mann-Whitney U test for quantitative variables and chi-square test or Fisher's exact test for categorical variables, as appropriate.

The correlation between NT-proBNP levels at 3 and 6 months and hemodynamic outcomes was assessed using Pearson's correlation coefficient. To analyze the effect of NT-proBNP levels separately at 3 and 6 months on each outcome of interest, we employed Cox proportional hazards models for all-cause mortality and the composite of all-cause mortality or pump thrombosis, with results presented as hazard ratios and 95% confidence intervals. A logistic regression model was used for the composite of all-cause mortality or RV failure, since time-to-event data were not available, with results presented as odds ratios and 95% confidence intervals.

To assess the impact of the longitudinal evolution of repeated NT-proBNP measurements, we implemented time-varying Cox proportional hazards models for all-cause mortality and the composite of all-cause mortality or pump thrombosis. For the composite of all-cause mortality or RV failure, a generalized linear mixed-effects model was employed to account for repeated measurements and missing data patterns.

According to the Prognosis Research Strategy guidelines,^12^ which advocate for a clinically hypothesis-driven approach for a priori selection of model variables, all hazard ratios and odds ratios were adjusted for the following covariates: age, sex, body mass index (BMI), history of implantable cardioverter defibrillator (ICD), history of CKD, ischemic cardiomyopathy, and the use of acute mechanical circulatory support (aMCS) before LVAD implantation.

Lastly, a multivariable logistic regression model with 3-knots restricted cubic spline functions and threshold effects, adjusted for the same covariates as mentioned above, was performed in the overall population to determine the optimal predictive cut-off point for 6-month NT-proBNP and to assess the shape of the associations between NT-proBNP levels (as a continuous measure) and the composite of all-cause mortality or RV failure.

All tests were 2-tailed. The value of *p* ≤ 0.05 was accepted as statistically significant. Analyses were performed using R software [version 4.3.2 (October 31, 2023)].

### Ethics

According to French ethics and regulatory law (Public Health Code), all patients received information about anonymized data collection. Written informed consent for participation was not required for this study in accordance with the national legislation. The study was registered by the Toulouse University Hospital (registration number: RnIPH 2025-57) and covered by the MR-004.

## Results

### Baseline characteristics

**Figure 1:**
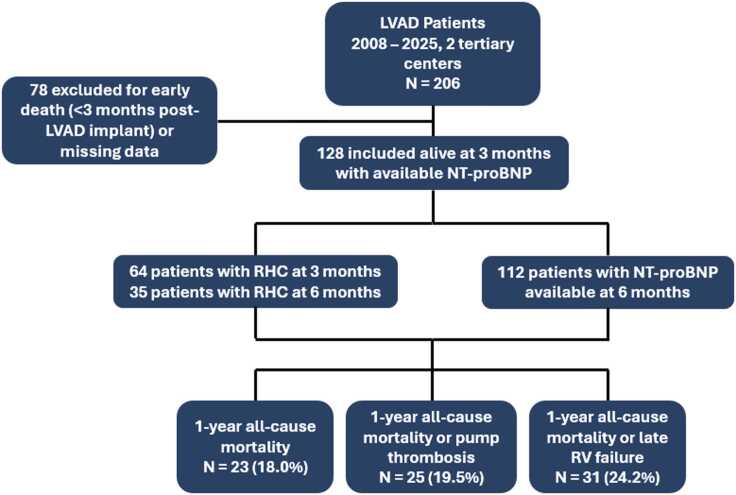
Flowchart of the study. LVAD, left ventricular assist device; NT-proBNP, N-terminal fragment of the brain natriuretic peptide; RHC, right heart catheterization; RV, right ventricular.

Among 206 patients who underwent LVAD implantation between 2008 and 2025 in our 2 institutions, 128 were included in the primary analysis (78 were excluded due to death within the first 3 postoperative months or missing data) (Figure 1).

In the overall cohort, the median age was 66.0 years (58.8-72.0), and 103 patients (80.5%) were male (Table 1). The most frequently implanted LVAD was the HeartMate 3 (*n* = 78, 60.9%), followed by the HeartMate II (*n* = 45, 35.2%) and the HeartWare (*n* = 5, 3.9%). The primary indications for LVAD implantation were BTT (53.9%) and DT (37.5%). Ischemic cardiomyopathy was the most common underlying HF etiology, accounting for 102 patients (79.7%). A total of 38 patients (29.7%) received aMCS before LVAD implantation, mainly with veno-arterial extracorporeal membrane oxygenation (*n* = 23, 60.5%). Cardiac resynchronization therapy and ICDs were present in 21.9% and 53.9% of patients, respectively. At 3 months, the median LVEF was 20.0% (15.0-29.5). Median peak VO₂ was 12.5 ml/min/kg (10.6-14.8), with a CIx of 2.5 liter/min/m² (2.1-2.7) and a PAPI of 1.8 (1.2-2.5) (Table 2). Comparison between 1-year survivors and nonsurvivors revealed no major differences in baseline characteristics, including age, preimplant INTERMACS classification, cardiomyopathy subtype and duration, or preoperative use of aMCS. Similarly, New York Heart Association class, LVEF, peak VO₂, 6MWT, and renal function assessed at 3 months postimplantation did not differ significantly between groups. Lastly, 3-month NT-proBNP levels were also similar between survivors and nonsurvivors, with median values of 1,141.0.0 pg/ml and 1,611.0 pg/ml, respectively (*p* = 0.22).

**Table 1:** Baseline Characteristics

Parameters or Characteristics	Overall population (*n* = 128)	1-year survivors (*n* = 109)	1-year nonsurvivors (*n* = 19)	*p*-value
Age, median (IQR), years	66.0 (58.8-72.0)	66.0 (59.0-73.0)	64.0 (57.0-70.5)	0.61
Male sex, *n* (%)	103 (80.5)	88 (80.7)	15 (78.9)	1.00
BMI, median (IQR), kg/m²	25.5 (23.6-28.9)	25.5 (23.5-28.9)	26.5 (24.2-29.4)	0.31
Cardiovascular risk factors, *n* (%)				
Hypertension Diabetes mellitus Family history Current smoking Dyslipidemia	48 (37.5) 31 (24.2) 27 (21.1) 35 (27.3) 66 (51.6)	41 (37.6) 25 (22.9) 22 (20.2) 29 (26.6) 56 (51.4)	7 (36.8) 6 (31.6) 5 (26.3) 6 (31.6) 10 (52.6)	1.00 0.40 0.55 0.87 1.00
Comorbidities, *n* (%)				
COPD CKD Stroke GERD IBD Peptic ulcer GI bleeding Colorectal cancer Malignant hemopathy Coagulation disorders Venous thromboembolism	15 (11.7) 31 (24.2) 19 (14.8) 4 (3.1) 1 (0.8) 4 (3.1) 4 (3.1) 1 (0.8) 1 (0.8) 1 (0.8) 11 (8.6)	12 (11.0) 27 (24.8) 15 (13.8) 4 (3.7) 1 (0.9) 4 (3.7) 3 (2.8) 1 (0.9) 1 (0.9) 1 (0.9) 10 (9.2)	3 (15.8) 4 (21.1) 4 (21.1) 0 (0.0) 0 (0.0) 0 (0.0) 1 (5.3) 0 (0.0) 0 (0.0) 0 (0.0) 1 (5.3)	0.47 1.00 0.48 1.00 1.00 1.00 1.00 1.00 1.00 1.00 1.00
Rhythm disorders, *n* (%)				
Atrial fibrillation CRT ICD	37 (28.9) 28 (21.9) 69 (53.9)	32 (29.4) 24 (22.0) 59 (54.1)	5 (26.3) 4 (21.1) 10 (52.6)	1.00 1.00 1.00
Previous CM, *n* (%)				1.00
Ischemic Dilated Other	102 (79.7) 24 (18.8) 2 (1.6)	86 (78.9) 21 (19.3) 2 (1.8)	16 (84.2) 3 (15.8) 0 (0.0)	
Duration of the CM, *n* (%)				0.95
<3 months 3 months-1 year 1-5 years >5 years	47 (36.7) 11 (8.6) 19 (14.8) 51 (39.8)	39 (35.8) 10 (9.2) 17 (15.6) 43 (39.4)	8 (42.1) 1 (5.3) 2 (10.5) 8 (42.1)	
aMCS before LVAD implant, *n* (%)				0.53
Overall V-A ECMO Impella ECMELLA	38 (29.7) 23 (60.5) 13 (34.2) 2 (5.3)	34 (31.2) 20 (58.8) 13 (38.2) 1 (2.9)	4 (21.1) 3 (75.0) 0 (0.0) 1 (25.0)	
INTERMACS stage at implantation, *n* (%)				0.39
1 2 3 4 5	22 (17.7) (*n* = 124) 13 (10.5) (*n* = 124) 30 (24.2) (*n* = 124) 45 (36.3) (*n* = 124) 14 (11.3) (*n* = 124)	20 (18.9) (*n* = 106) 12 (11.3) (*n* = 106) 24 (22.6) (*n* = 106) 40 (37.7) (*n* = 106) 10 (9.4) (*n* = 106)	2 (11.1) (*n* = 18) 1 (5.6) (*n* = 18) 6 (33.3) (*n* = 18) 5 (27.8) (*n* = 18) 4 (22.2) (*n* = 18)	
LVAD device, *n* (%)				0.56
Heartmate 2 Heartmate 3 Heartware	45 (35.2) 78 (60.9) 5 (3.9)	39 (35.8) 65 (59.6) 5 (4.6)	6 (31.6) 13 (68.4) 0 (0.0)	
Indication for implantation, *n* (%)				0.96
Bridge to transplant Destination therapy Bridge to decision Bridge to candidacy	69 (53.9) 48 (37.5) 6 (4.7) 5 (3.9)	59 (54.1) 40 (36.7) 5 (4.6) 5 (4.6)	10 (52.6) 8 (42.1) 1 (5.3) 0 (0.0)	
Treatment at 3 months postimplantation, *n* (%)				
Antiplatelet therapy Anticoagulant Betablocker ACE-I or ARB ARNi ARM iSGLT2	104 (84.6) (*n* = 123) 124 (100.0) (*n* = 124) 84 (67.7) (*n* = 124) 68 (54.8) (*n* = 124) 3 (2.4) (*n* = 123) 61 (49.2) (*n* = 124) 19 (15.3) (*n* = 124)	89 (84.8) (*n* = 105) 106 (100.0) (*n* = 106) 75 (70.8) (*n* = 106) 58 (54.7) (*n* = 106) 2 (1.9) (*n* = 105) 51 (48.1) (*n* = 106) 12 (11.3) (*n* = 106)	15 (83.3) (*n* = 18) 18 (100.0) (*n* = 18) 12 (66.7) (*n* = 18) 10 (55.6) (*n* = 18) 1 (5.6) (*n* = 18) 10 (55.6) (*n* = 18) 7 (38.9) (*n* = 18)	1.00 1.00 0.94 1.00 0.38 0.74 < 0.01

Abbreviations: ACE-I, angiotensin-converting enzyme; aMCS, acute mechanical circulatory support; ARB, angiotensin II receptor blockers; ARM, aldosterone receptor antagonists; ARNi, angiotensin receptor neprilysin inhibitor; BMI, body mass index; CKD, chronic kidney disease; CM, cardiomyopathy; COPD, chronic obstructive pulmonary disease; CRT, cardiac resynchronization therapy; GERD, gastroesophageal reflux disease; GI, gastrointestinal; IBD, inflammatory bowel disease; ICD, implantable cardioverter defibrillator; IQR, interquartile range; iSGLT2, sodium-glucose cotransporter type 2 inhibitors; LVAD, left ventricular assist devices; V-A ECMO, veno-arterial extracorporeal membrane oxygenation.

**Table 2:** Functional, Biological, and Hemodynamic Assessment 3 Months After LVAD Implantation

Parameters or Characteristics	Overall population (*n* = 128)	1-year survivors (*n* = 109)	1-year nonsurvivors (*n* = 19)	*p*-value
Functional assessment at 3 months				
NYHA ≥ 3, *n* (%) Peak VO_2_, median (IQR), ml/min/kg 6MWT, median (IQR), m	7 (5.9) (*n* = 118) 12.5 (10.6-14.8) (*n* = 55) 400.0 (344.0-450.0) (*n* = 33)	6 (5.9) (102) 12.4 (10.4-14.4) (*n* = 50) 402.5 (336.3-465.0) (*n* = 30)	1 (6.3) (*n* = 16) 12.7 (12.6-15.9) (*n* = 5) 345.0 (344.5-382.5) (*n* = 3)	1.00 0.24 0.56
Blood tests at 3 months, median (IQR)				
NT-proBNP, pg/ml Creatinine, μmol/liter eGFR, ml/min/1.73 m² Albumin, g/liter Bilirubin, mg/liter Hemoglobin, g/dl Platelets, g/liter	1,178.5 (527.0-1,733.3) 83.0 (71.8-105.0) (*n* = 124) 82.0 (62.0-95.0) (*n* = 105) 34.0 (31.5-38.0) (*n* = 67) 8.0 (5.0-11.0) (*n* = 99) 12.0 (11.0-12.4) (*n* = 125) 264.0 (209.0-309.5) (*n* = 115)	1,141.0 (504.0-1,663.0) 83.0 (71.0-105.0) (*n* = 105) 82.5 (64.5-96.0) (*n* = 88) 34.5 (32.0-38.3) (*n* = 56) 8.0 (5.0-11.0) (*n* = 84) 11.9 (11.0-12.4) (*n* = 106) 269.0 (214.0-322.0) (*n* = 97)	1,611.0 (838.0-2,087.0) 80.0 (72.5-112.0) 76.0 (57.0-93.0) (*n* = 17) 31.0 (28.0-36.5) (*n* = 11) 8.2 (5.7-10.2) (*n* = 15) 12.0 (10.2-12.3) 247.0 (183.8-271.0) (*n* = 18)	0.22 0.82 0.31 0.24 0.62 0.92 0.04
Echocardiography parameters at 3 months				
LVEF, median (IQR), % TAPSE, median (IQR), mm PSVtdi, median (IQR), cm/s	20.0 (15.0-29.5) (*n* = 62) 14.0 (11.0-16.0) (*n* = 73) 7.0 (6.0-8.0) (*n* = 64)	20.0 (15.0-26.5) (*n* = 55) 14.0 (11.0-15.0) (*n* = 62) 7.0 (6.0-8.0) (*n* = 55)	20.0 (15.0-30.0) (*n* = 7) 15.0 (12.0-16.0) (*n* = 11) 6.0 (5.0-8.0) (*n* = 9)	0.81 0.57 0.50
Hemodynamic measurements in right heart catheterization at 3 months, median (IQR)				
RAP, mm Hg sPAP, mm Hg dPAP, mm Hg mPAP, mm Hg PCWP, mm Hg RAP/PCWP PAPI Cardiac output, liter/min Cardiac index, liter/min/m²	7.0 (5.0-9.5) (*n* = 63) 25.0 (22.0-30.0) (*n* = 57) 10.0 (7.0-13.0) (*n* = 56) 15.0 (15.0-19.3) (*n* = 64) 9.0 (7.0-12.0) (*n* = 64) 0.86 (0.69-1.09) (*n* = 64) 1.8 (1.2-2.5) (*n* = 64) 4.7 (4.0-5.0) (*n* = 64) 2.5 (2.1-2.7) (*n* = 64)	7.0 (5.0-9.5) (*n* = 51) 25.0 (21.3-31.5) (*n* = 46) 9.5 (7.3-11.8) (*n* = 46) 14.5 (12.0-19.3) (*n* = 52) 9.5 (7.0-12.0) (*n* = 52) 0.83 (0.66-1.06) (*n* = 52) 1.8 (1.2-2.3) (*n* = 52) 4.7 (4.1-5.0) (*n* = 52) 2.4 (2.2-2.7) (*n* = 52)	8.5 (5.8-9.3) (*n* = 12) 26.0 (23.0-29.5) (*n* = 11) 10.0 (7.3-13.4) (*n* = 10) 25.5 (12.3-19.3) (*n* = 12) 8.5 (7.5-10.6) (*n* = 12) 1.0 (0.86-1.11) (*n* = 12) 1.8 (1.1-2.5) (*n* = 12) 4.6 (3.8-5.1) (*n* = 12) 2.5 (2.1-2.8) (*n* = 12)	0.59 0.92 0.65 0.76 0.53 0.25 0.92 0.89 0.72

Abbreviations: 6MWT, 6-minute walk test; dPAP, diastolic pulmonary arterial pressure; eGFR, estimated glomerular filtration rate; IQR, interquartile ranges; LVAD, left ventricular assist devices; LVEF, left ventricular ejection fraction; mPAP, mean pulmonary arterial pressure; NT-proBNP, N-terminal fragment of the brain natriuretic peptide; NYHA, New York Heart Association; PAPI, pulmonary artery pulsatility index; PCWP, pulmonary capillary wedge pressure; PSVtdi, peak systolic S′ wave tricuspid annular velocity; RAP, right atrial pressure; sPAP, systolic pulmonary arterial pressure; TAPSE, tricuspid annular plane systolic excursion.

**Figure 2:**
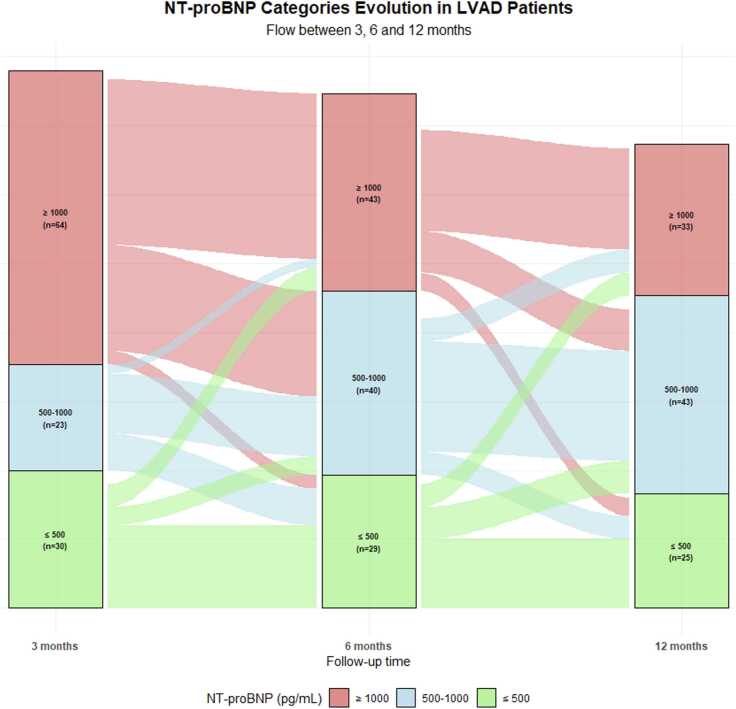
Alluvial plot illustrating NT-proBNP trajectories at 3, 6, and 12 months following LVAD implantation. LVAD, left ventricular assist device; NT-proBNP, N-terminal fragment of the brain natriuretic peptide.

NT-proBNP categories demonstrated progressive improvement over the follow-up period, with a notable reduction in the proportion of patients with NT-proBNP ≥1,000 pg/ml from 3 to 12 months post-LVAD implantation (Figure 2). This temporal evolution was accompanied by a corresponding increase in patients achieving intermediate (500-1,000 pg/ml) and lower NT-proBNP categories (≤500 pg/ml).

### Correlation between NT-proBNP and hemodynamic parameters

**Figure 3:**
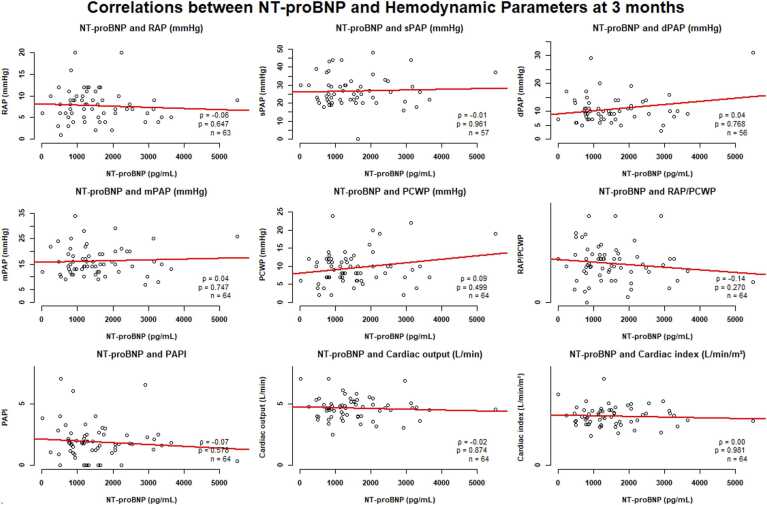
Correlations between NT-proBNP and hemodynamic parameters at 3 months. dPAP, diastolic pulmonary artery pressure; LVAD, left ventricular assist device; mPAP, mean pulmonary artery pressure; NT-proBNP, N-terminal fragment of the brain natriuretic peptide; PAPI, pulmonary artery pulsatility index; PCWP, pulmonary capillary wedge pressure; RAP, right atrial pressure; sPAP, systolic pulmonary artery pressure.

At 3 months, the correlation analysis between NT-proBNP levels and hemodynamic parameters post-LVAD implantation revealed no statistically significant associations across all measured variables (Figure 3). Despite the wide range of NT-proBNP values (0-5,000 pg/ml), none of the hemodynamic parameters, including RAP, sPAP/mPAP/dPAP, PCWP, or cardiac output/index, showed meaningful correlations with NT-proBNP levels (all *p*-values >0.05).

**Figure 4:**
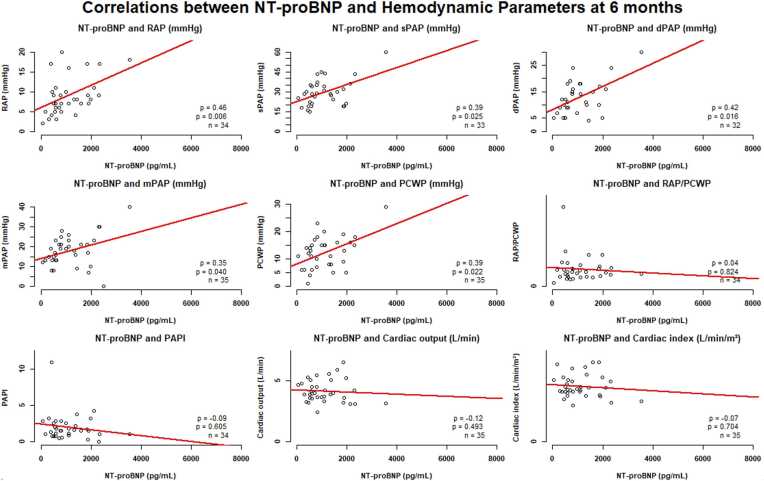
Correlations between NT-proBNP and hemodynamic parameters at 6 months. dPAP, diastolic pulmonary artery pressure; LVAD, left ventricular assist device; mPAP, mean pulmonary artery pressure; NT-proBNP, N-terminal fragment of the brain natriuretic peptide; PAPI, pulmonary artery pulsatility index; PCWP, pulmonary capillary wedge pressure; RAP, right atrial pressure; sPAP, systolic pulmonary artery pressure.

Conversely, at 6 months, the correlation analysis demonstrated several statistically significant moderate positive associations between NT-proBNP levels and RAP (ρ = 0.46, *p* < 0.01), sPAP (ρ = 0.39, *p* = 0.03), dPAP (ρ = 0.42, *p* = 0.02), and PCWP (ρ = 0.39, *p* = 0.02) (Figure 4). In contrast, cardiac output/index and PAPI showed no significant correlations with NT-proBNP levels at this time point.

### Correlation between NT-proBNP and 1-year clinical outcomes

**Figure 5:**
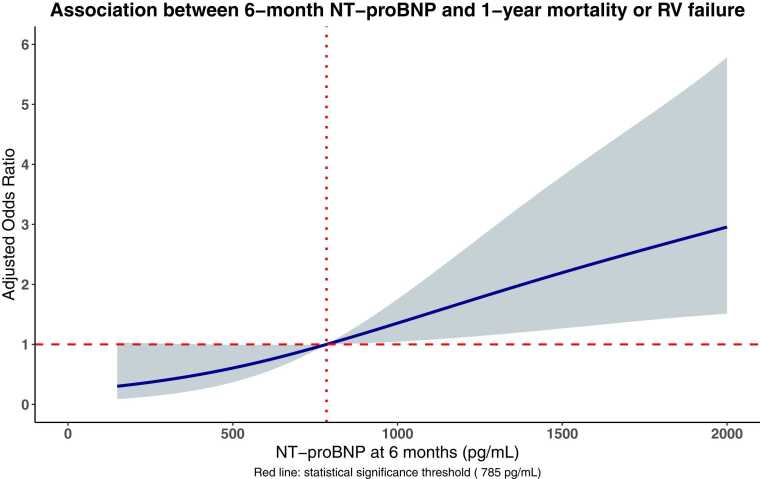
Restricted cubic spline curves for the relationship between NT-proBNP and all-cause mortality. The blue area represents the 95% confidence interval. NT-proBNP, N-terminal fragment of the brain natriuretic peptide; RV, right ventricular.

After 1 year of follow-up, 18 patients (18.0%) had died, 25 (19.5%) experienced either death or pump thrombosis, and 31 (24.2%) had died or developed RV failure. While NT-proBNP at 3 months showed no statistically significant associations with any of the composite outcomes at 1-year follow-up, NT-proBNP levels at 6 months emerged as a strong independent predictor of adverse events (Table 3). Specifically, each 500 pg/ml increase in NT-proBNP at 6 months was associated with significantly increased risks of all-cause mortality or pump thrombosis (adjusted hazard ratio 1.34 [1.07-1.67]) and all-cause mortality or RV failure (adjusted odds ratio 1.53 [1.17-2.01]). Interestingly, the use of repeated NT-proBNP measurements at both 3 and 6 months did not enhance the prognostic discrimination compared to single 6-month measurements. A monotonic relationship between 6-month NT-proBNP level and the composite of all-cause mortality or RV failure was confirmed by spline analysis results, as shown in Figure 5. The optimal cut-off for 6-month NT-proBNP predicting 1-year all-cause mortality or RV failure was 785 pg/ml.

**Table 3:** Primary and Secondary Outcomes at 1 Year

Parameters or Characteristics	NT-proBNP (pg/ml)	aHR or aOR^a^ for each outcome of interest at 1 year (95% CI)
NT-proBNP at 3 months		
All-cause mortality	+ 200	1.03 (0.96-1.11)
	+ 500	1.09 (0.90-1.31)
	+ 1,000	1.18 (0.81-1.72)
All-cause mortality or pump thrombosis	+ 200	1.06 (0.99-1.14)
	+ 500	1.16 (0.96-1.40)
	+ 1,000	1.35 (0.93-1.96)
All-cause mortality or right ventricular failure	+ 200	1.09 (0.99-1.19)
	+ 500	1.23 (0.98-1.54)
	+ 1,000	1.51 (0.97-2.37)
NT-proBNP at 6 months		
All-cause mortality	+ 200	1.08 (0.99-1.17)
	+ 500	1.20 (0.97-1.49)
	+ 1,000	1.45 (0.94-2.23)
All-cause mortality or pump thrombosis	+ 200	1.12 (1.03-1.23)
	+ 500	1.34 (1.07-1.67)
	+ 1,000	1.80 (1.15-2.80)
All-cause mortality or right ventricular failure	+ 200	1.19 (1.07-1.32)
	+ 500	1.53 (1.17-2.01)
	+ 1,000	2.35 (1.37-4.03)
Repeated NT-proBNP measurements at 3 and 6 months		
All-cause mortality	+ 200	1.05 (0.96-1.14)
	+ 500	1.12 (0.90-1.39)
	+ 1,000	1.25 (0.81-1.92)
All-cause mortality or pump thrombosis	+ 200	1.05 (0.97-1.14)
	+ 500	1.14 (0.93-1.40)
	+ 1,000	1.30 (0.87-1.95)
All-cause mortality or right ventricular failure	+ 200	1.09 (0.78-1.53)
	+ 500	1.24 (0.53-2.87)
	+ 1,000	1.53 (0.29-8.24)

Abbreviations: aHR, adjusted hazard ratio; aMCS, acute mechanical circulatory support; aOR, adjusted odds ratio; BMI, body mass index; CI, confidence interval; CKD, chronic kidney disease; ICD, implantable cardioverter defibrillator; LVAD, left ventricular assist device; NT-proBNP, N-terminal fragment of the brain natriuretic peptide.

All-cause mortality and the composite outcome of all-cause mortality or pump thrombosis are presented as hazard ratios. The composite outcome of all-cause mortality or right ventricular failure is presented as odds ratios.

a Adjusted for age, sex ratio, BMI, history of ICD, history of CKD, ischemic cardiomyopathy, and the need for aMCS before LVAD implant.

## Discussion

To date, this study represents the largest analysis evaluating the prognostic value of NT-proBNP in patients with LVAD. Our main findings are as follows: (1) overall, NT-proBNP levels tend to progressively decrease after implantation; (2) NT-proBNP measured at 3 months postimplantation showed no correlation with hemodynamic parameters or clinical outcomes at 1 year, including all-cause mortality, RV failure, or pump thrombosis; and (3) in contrast, NT-proBNP at 6 months was significantly correlated with pressure-related hemodynamic parameters (RAP, sPAP, dPAP, mPAP, and PCWP), though not with cardiac output/index, and was also associated with clinical outcomes such as all-cause mortality, pump thrombosis, and RV failure.

In HF patients without chronic mechanical circulatory support, NT-proBNP is generally considered a relatively reliable marker of the degree of HF compensation. Its variations are usually well correlated with hemodynamic indices, including pressure parameters (RAP, sPAP, dPAP, mPAP, and PCWP) and cardiac output/index. In a prospective study of 40 intensive care unit patients with native hearts, Forfia et al^11^ demonstrated significant positive correlations between NT-proBNP and RAP (ρ = 0.56, *p* < 0.04), mPAP (ρ = 0.66, *p* = 0.01), and PCWP (ρ = 0.73, *p* < 0.01), as well as a negative correlation with cardiac index (ρ = −0.71, *p* < 0.01), in the absence of CKD. Similar findings have been reported in patients with established chronic HF due to ischemic or idiopathic dilated cardiomyopathy, where moderate but significant correlations were observed between natriuretic peptides and both mPAP (ρ = 0.45, *p* < 0.01) and PCWP (ρ = 0.46, *p* < 0.01).^13^ In contrast, our findings in LVAD recipients revealed no significant correlation between NT-proBNP and hemodynamic parameters at 3 months—neither for pressure nor flow-related variables. However, at 6 months, NT-proBNP was significantly associated with pressure parameters but remained unrelated to cardiac output/index, although correlation coefficients were generally lower than those observed in native hearts. Several hypotheses may account for this discrepancy. First, LVAD-induced left ventricular remodeling may fundamentally alter the traditional link between NT-proBNP and hemodynamic. The progressive unloading of the left ventricle reduces myocardial wall stress, which is a primary trigger for natriuretic peptide release, thereby potentially disrupting the peptide’s relationship with conventional filling pressures. Second, while NT-proBNP levels typically decrease after LVAD implantation in parallel with improved hemodynamic,^14^ they often remain elevated compared to those in chronic HF patients without mechanical support.^15^ This persistent elevation may reflect residual myocardial pathology at the cellular level, despite mechanical unloading. Third, RV adaptation to the altered loading conditions introduced by LVAD support likely requires several months to stabilize, which may explain the lack of correlation between NT-proBNP and hemodynamic indices observed at 3 months. Fourth, the continuous, nonpulsatile flow generated by most contemporary LVADs may disrupt normal mechanotransduction pathways involved in natriuretic peptide secretion, and may also influence neurohormonal activation—including sympathetic and renin-angiotensin-aldosterone system pathways^15^—which in turn could alter NT-proBNP dynamics. Lastly, the heterogeneity in residual native heart function (notably RV function) and aortic valve opening among patients may lead to complex and individualized hemodynamic profiles that further obscure straightforward associations between NT-proBNP and standard pressure or flow parameters. Collectively, these factors suggest that, in LVAD recipients, NT-proBNP may reflect overall disease severity or residual pathophysiological burden rather than directly mirroring hemodynamic status at a given time point. Consistently, in contrast to the 3-month measurement, NT-proBNP at 6 months was also well correlated with clinical outcomes of all-cause mortality, pump thrombosis, or RV failure, suggesting that the 6-month time point may represent an optimal window for risk stratification in LVAD patients, potentially reflecting the stabilization period following device implantation and the emergence of longer-term complications. Overall, the transition between 3 and 6 months may represent a period in which postoperative effects diminish, and hemodynamic parameters regain a stronger predictive value for biomarker levels. Accordingly, our findings suggest that beyond the 3-month mark following LVAD implantation, NT-proBNP may serve as a simple and widely accessible surrogate marker of progressive myocardial dysfunction and/or lack of HF stabilization under LVAD support, potentially indicating the need to accelerate bridging to transplant in selected patients. Elevated levels could act as an early warning signal, prompting timely evaluation by the advanced HF team to investigate potential early signs of decompensation, optimize therapy, and ultimately prevent avoidable hospitalizations. In this perspective, our results align with those reported for preimplant NT-proBNP, which was also found to be significantly associated with 1-month all-cause mortality^16^ and the ventricular arrhythmias.^17^ Interestingly, in a previous study including 127 ambulatory LVAD patients, an increase in NT-proBNP from 1 to 3 months was associated with a higher risk of HF hospitalization and death during a median follow-up of 17 months.^18^ However, this is not entirely comparable to our findings, as we chose to exclude patients who died within the first 3 months after LVAD implantation, considering this period to reflect early postoperative vulnerability, where mortality may result from immediate perioperative complications.

## Limitations

First, this study is limited by its retrospective design, which may have introduced biases in data completeness. Moreover, our results may have been influenced by the relatively small sample size, potentially leading to limited statistical power. Furthermore, although multicenter, the analysis was restricted to 2 geographically close French centers, and the population was heterogeneous due to inclusion over a 17-year period during which significant changes occurred in device technology (Heartmate II, Heartware, and Heartmate 3), biomarker assay methods, and treatment strategies. Additionally, the occurrence of HF rehospitalizations was not systematically collected, although this outcome could have provided valuable insights into the prognostic relevance of NT-proBNP levels. Beyond clinical outcomes, it would also have been valuable to analyze the correlation between NT-proBNP trajectories and functional parameters such as the 6MWT or repeated measurements of peak oxygen uptake. Furthermore, extending NT-proBNP monitoring with repeated measurements and prolonging follow-up beyond 1 year would be essential to better understand the long-term prognostic value of this biomarker and its potential role in guiding ongoing management strategies. Finally, we lacked sufficient data to assess the potential role of NT-proBNP in predicting myocardial recovery or the progression of left ventricular remodeling—an area of interest given the currently conflicting findings in the literature.^19^, ^20^, ^21^

## Conclusion

In this multicenter retrospective study, NT-proBNP measured at 6 months post-LVAD implantation is a significant predictor of adverse clinical and hemodynamic outcomes at 1 year, supporting its role in patients’ monitoring and risk stratification. Prospective studies are warranted to validate these findings.

## Author contribution

**Miloud Cherbi:** Design of the study, statistical analysis, analysis and interpretation of the data, drafting the first manuscript, and final approval of the version to be submitted. **Joaquim Verdaguer:** Data collection, analysis and interpretation of the data, and final approval of the version to be submitted. **Romain Itier:** Design of the study, analysis and interpretation of the data, and final approval of the version to be submitted. **Laurence Barde:** Analysis and interpretation of the data and final approval of the version to be submitted. **Pauline Fournier:** Data collection and final approval of the version to be submitted. **Etienne Grunenwald:** Data collection and final approval of the version to be submitted. **Philippe Gaudard:** Data collection and final approval of the version to be submitted. **Philippe Rouvière:** Data collection and final approval of the version to be submitted. **Adrien Molina:** Data collection, analysis of the data, and final approval of the version to be submitted. **Aurore Ughetto:** Revising critically for important intellectual content of the manuscript and final approval of the version to be submitted. **Clément Delmas:** Design of the study, analysis and interpretation of the data, coordination of the research, revising critically for important intellectual content of the manuscript, and final approval of the version to be submitted.

## Disclosure statement

C.D. reports consulting and lectures fees from ABOTT. The other authors declare that they have no known competing financial interests or personal relationships that could have appeared to influence the work reported in this paper.

The authors acknowledge all the medical and surgical teams in our 2 centers involved in the management of this severe population of advanced heart failure patients.

Funding: None.
